# Zero as a semantic boundary: Rethinking the mental number line

**DOI:** 10.1007/s00426-026-02301-w

**Published:** 2026-04-28

**Authors:** Tali Leibovich-Raveh, Christine Mireb-Geraisy

**Affiliations:** Department of Mathematics Education, University of Haifa, Haifa, Israel

## Abstract

Zero poses a unique challenge for numerical cognition because it denotes absence in cardinal contexts, yet functions as a formal entity in ordinal or interval systems. We examined how adults compare zero to positive single-digit numbers across symbolic, nonsymbolic, and mixed formats. Results replicated classic distance and end effects for positive numbers. However, the cognitive status of zero was found to be strictly format-dependent. In the symbolic format, the distance effect was driven by boundary values (0 and 1) and vanished when they were removed, suggesting symbolic zero functions as a structural anchor that defines the semantic transition from absence to quantity. In contrast, nonsymbolic zero was integrated into a continuous, nonlinear magnitude gradient, following the same psychophysical power-function pattern as other quantities. Furthermore, the [0, 1] pair elicited a unique facilitation in nonsymbolic and mixed formats, but not in the symbolic format. Together, these findings suggest that zero is not merely a point on a scale but a multi-faceted construct: while nonsymbolic zero is processed as a perceptual category of absence, symbolic zero acts as a semantic boundary that redefines the internal structure of the mental number line.

## Introduction

Understanding how humans mentally represent and compare numerical magnitudes is fundamental to the study of numerical cognition. Traditionally, this representation has been conceptualized as a spatial “mental number line” (MNL), supported by robust behavioral markers such as the distance effect, where comparisons become faster and more accurate as the numerical gap increases (Moyer & Landauer, 1967), and the end effect, the facilitated processing of values at the boundaries of a range, such as 1 (Banks, 1977). However, contemporary models emphasize that these effects may not reflect a literal spatial map, but rather a dynamic system of relative-magnitude and semantic processes that can, but need not, rely on a spatially organized continuum (Krajcsi & Kojouharova, 2017).

Within this framework, zero presents a unique cognitive challenge because its meaning is fundamentally multi-faceted. In cardinal contexts, zero denotes “nothingness” or the absolute absence of quantity; conversely, in ordinal or interval contexts, such as floor levels or temperature scales, it functions as a formal numerical entity or a fixed reference point. Previous works indicate that these roles may evoke qualitatively distinct processing mechanisms, where zero functions either as a categorical boundary between “none” and “some” or as a graded magnitude integrated into the MNL (Nieder, 2016; Pinhas & Tzelgov, 2012). By distinguishing between these roles, we can better understand how zero is integrated into the broader numerical system. To test this integration, we rely on the aforementioned behavioral markers: the distance effect and the end effect.

Crucially, these behavioral markers do not operate in a vacuum; their manifestation is highly sensitive to the representational format in which numbers are presented. Symbolic numbers (e.g., Arabic digits) and nonsymbolic quantities (e.g., sets of items) are known to engage partly distinct processing systems (Lyons et al., 2012). This distinction may be especially pronounced for zero, whose nonsymbolic representation, the absence of items, is inherently abstract.

Against this background, the present study examines how adults compare zero to positive single-digit numbers across symbolic and nonsymbolic formats, and whether classic magnitude effects emerge similarly across conditions. This approach allows us to test whether zero is treated like other numbers or functions as a conceptual boundary at the edge of the MNL. In line with contemporary perspectives, we refer to the MNL as a useful descriptive metaphor rather than a fixed spatial map of numerical representation.

### The distance and end effects

Understanding how humans mentally represent and compare numerical magnitudes is central to the study of numerical cognition (Gilmore et al., 2010). In this context, classic effects that inform us about the cognitive representation of the numerical system are the distance effect and the end effect. The distance effect was first reported by Moyer and Landauer (1967). In their study adults were asked to decide which of two single-digit numbers was larger in value. Plotting RT and accuracy as a function of the difference between the value of the digits, the results demonstrated that RT and error rates decrease as the numerical distance between the numerals increases. Since then, other studies have replicated this pattern showing a similar trend in magnitude for both comparison tasks and same/different tasks, consistently showing the robust nature of the distance effect (Brysbaert, 1995; Dehaene, 1996; Dehaene et al., 1990; Van Opstal & Verguts, 2011).

The end effect was first investigated by Banks (1977). Participants compared pairs of numbers with or without specific boundary values. In this study, RT to pairs with boundary values was shorter compared with RT to pairs without boundary values. The author suggested that end numbers are tagged by semantic memory as the smallest ones, i.e., especially the lower boundary number (1), enhancing RTs. Leth-Steensen and Marley (2000) provided converging evidence using a different paradigm. They asked adult participants to decide which image of a man in a pair was taller, with men selected from a group of six men, to ensure the presence of boundary items (the shortest and the tallest). Their analysis revealed a significant main effect of end position. The authors concluded that items at the ends of an ordered sequence have special status in memory and processing, which affects people’s decisions in comparison tasks. Both Banks’ numerical comparison task and Leth-Steensen and Marley’s height comparison task support the phenomenon that boundary items are processed differently than middle-range items.

In practice, the end effect means that RT tends to be reduced in pairs with end numbers, particularly when the end number is 1. Research has predominantly investigated this lower boundary effect (Pinhas & Tzelgov, 2012), with fewer studies examining upper boundary values. Recent work by Lozin and Pinhas (2025) explored whether both lower and upper end effects occur across different numerical ranges. Adult participants were asked to compare pairs of numbers. Each participant was randomly assigned to one of six conditions that varied the highest number in the range (10, 100, 1,000, 10,000, 100,000, 1,000,000), while the compared number was always a single-digit number ranging from 1 to 9. The comparison tasks included pairs with lower end value, upper end value and non-end values. The results revealed a significant end effect for both the smallest and the largest numbers in each range, supporting Banks’s (1977) model of boundary values by demonstrating that participants assign special semantic status in memory to clearly defined boundary values.

Overall, the distance and end effects provide robust behavioral markers of magnitude processing. Yet, current models increasingly emphasize that these effects do not unambiguously index a spatial MNL. Instead, they emerge from dynamic interactions among semantic associations, task demands, and cognitive control processes that may underlie or modulate spatial–numerical mappings (Krajcsi & Kojouharova, 2017; Schliephake et al., 2021).

### The cognitive challenges of zero representation

As established in the introduction, the primary cognitive challenge of zero lies in its multi-faceted nature, where its meaning shifts between denoting absolute absence and serving as a formal numerical reference point. Unlike positive integers, which are grounded in the perception of discrete cardinal quantities, zero’s representation is highly context-dependent. This conceptual complexity arises because zero must be understood simultaneously as a categorical boundary, i.e., the qualitative transition between ‘none’ and ‘some’, and as a graded magnitude integrated into the MNL. Consequently, zero occupies a unique position as a numerical ‘origin’ that requires a higher level of abstract reasoning to reconcile its role as a ‘non-quantity’ with its function as a mathematical entity.

In addition to these conceptual difficulties, a significant methodological challenge in numerical cognition research involves how nonsymbolic zero is operationalized. Mostly, studies have represented nonsymbolic zero as an empty set—a completely blank display containing no visual elements (e.g., Barnett & Fleming, 2024; Gilmore et al., 2010; Merritt et al., 2009 and; Zaks-Ohayon et al., 2021, 2022). This approach, however, carries two significant limitations. First, multiple linguistic terms can denote an empty set, such as ‘none’ or ‘empty’, making the word ‘zero’ just one of several possible labels. Second, as demonstrated before, empty sets are not always intuitively processed as numerical magnitudes (Cohrssen et al., 2024; Hartmann et al., 2022; Nieder, 2016; Pinhas & Tzelgov, 2012). These limitations suggest that the numerical status of the empty set is not absolute but may emerge only under specific representational conditions.

Building on this notion that format and context are critical, Zaks-Ohayon et al. (2022) examined the conditions under which an empty set is perceived as a numerical entity. They distinguished between homogeneous pairs (where both numbers are in the same format, e.g., two dot arrays) and mixed-notation pairs (where a symbolic digit is compared to a nonsymbolic array). Their findings revealed a striking dissociation: while participants successfully integrated the empty set into a numerical scale during homogeneous comparisons, this integration failed in the mixed-notation condition. Although the symbolic zero retained its numerical status, the nonsymbolic empty set appeared to lose its numerical meaning when compared against a symbolic digit. This suggests that the numerical representation of ‘nothing’ is highly sensitive to the representational context.

The conceptual complexity of zero is further evidenced by its unique developmental trajectory, requiring more abstract reasoning than positive integers. Krajcsi and Kojouharova (2021) investigated this by testing 3- to 5-year-olds on ‘give-N’ tasks (e.g., ‘Give the monkey zero bananas’) alongside assessments of number knowledge and metaknowledge. While children succeeded in object-based tasks, they consistently struggled with verbal tasks involving the word ‘zero’. Notably, even operationally competent children often identified ‘one’ as the smallest number, suggesting that zero is not yet fully recognized as a numerical entity at these ages. These findings underscore the fundamental cognitive gap between understanding zero and positive integers.

Expanding on these developmental findings, Nieder (2016) proposed a four-stage framework for understanding how the empty set is integrated as a numerical quantity. Initially, the absence of a stimulus corresponds to inactive neural states without a specific representation. In the second stage, behavioral relevance emerges as ‘absence’ becomes a meaningful category through reinforcement learning, allowing individuals to distinguish ‘something’ from ‘nothing’. The critical third stage involves integrating this categorical representation into the quantitative system, where empty sets acquire numerical meaning and are positioned at the lowest end of the MNL. This stage requires a fundamental cognitive transformation: turning a non-quantitative absence into an abstract numerical magnitude. Finally, individuals develop symbolic reasoning and an understanding of number theory rules. According to this model, children begin incorporating empty set representations into their mental number line around the age of four.

### The role of zero in mathematics

Despite these cognitive challenges, zero plays a central role in formal numerical systems and mathematical operations. In mathematics, zero serves multiple essential functions: (a) as a placeholder in positional notation systems, (b) as the origin point in measurement scales, (c) as a representation of the cardinality of the empty set in set theory (which is often denoted by the symbol ∅ to distinguish it from the number zero), (d) as the symmetry point between positive and negative numbers on the number line, (e) as the additive identity element where any number plus zero equals that number (*n* + 0 = n), (f) as the element that yields the additive inverse when subtracted from any number (0 - n = -n), (g) as the absorbing element in multiplication where any quantity multiplied by zero equals zero (*n* × 0 = 0), (h) as the dividend in division operations where zero divided by a non-zero number equals zero (0 ÷ *n* = 0), although division by zero remains undefined (Barton, 2020). These diverse mathematical roles underscore zero’s fundamental importance in numerical reasoning and highlight the complexity underlying its cognitive representation.

Beyond its arithmetic functions, zero is fundamental to numerical reasoning and algebraic competence (Russell & Chernoff, 2011; Vest & Alibali, 2024; Wellman & Miller, 1986). Akdeniz et al. (2022) explored this by interviewing students (ages 10–12) with and without dyscalculia—a specific learning disorder characterized by persistent difficulties in understanding numbers and performing mathematical calculations—on their perception of zero. The study assessed diverse interpretations, including zero’s role as a placeholder, a numerical concept, and the cardinality of an empty set. While no significant differences emerged between the groups, findings revealed that students held inconsistent and often fragmented interpretations, sometimes viewing zero as an ‘ineffective’ number. The authors concluded that zero’s abstract nature can hinder mathematical comprehension, emphasizing that mastery requires students to explicitly bridge the concept of zero with the idea of ‘nullity’. These results suggest that students’ struggles stem from zero’s inherent conceptual complexity, necessitating a deeper, more integrated approach to its instruction.

Subsequent research has explored how zero comprehension strengthens mathematical skills, particularly in integer arithmetic. Vest and Alibali (2024) examined students aged 10–13 as they solved addition, subtraction, and word problems involving positive and negative numbers. The study evaluated participants’ grasp of zero as a symmetry point on the number line, a conceptual bridge between positive and negative values. Results revealed that students with a strong understanding of this symmetry demonstrated greater fluency in integer arithmetic and were significantly more likely to employ efficient strategies based on the additive inverse principle. These findings suggest that zero comprehension involves multiple cognitive dimensions that require explicit instructional attention to bridge conceptual gaps.

Together, these results indicate that zero is not just a basic numerical marker but a cognitively demanding construct, prompting further inquiry into how it is represented and processed in the mind.

### Cognitive representation of zero and positive whole numbers

Symbolic representations of natural numbers (e.g., Arabic numerals) and nonsymbolic quantities (e.g., sets of dots) are thought to engage different processing mechanisms. For example, Lyons et al. (2012) aimed to examine whether adults process symbolic and nonsymbolic positive numbers by the same cognitive system. They asked participants to compare, in different blocks, either two sets of dots, two Arabic numerals (i.e., pure conditions), or pairs that were comprised of a set of dots and a numeral (i.e., mixed condition). They hypothesized that if symbolic and nonsymbolic numbers rely on the same mechanism, RTs would follow a graded pattern: fastest for symbolic pairs, slower for mixed pairs, and slowest for nonsymbolic pairs. However, if symbolic and nonsymbolic numbers are processed by different mechanisms, then RT would be longest for the mixed condition, because of an extra step of translation between the two notations. Their findings supported a separate-mechanism account.

Following up on this study to gain more insight into the representation of zero, Zaks-Ohayon et al. (2022) investigated the conditions under which a null numerosity is perceived as a numerical entity on the MNL. Similar to Lyons et al., they employed magnitude comparison tasks in three blocks, using both pure and mixed sets. The authors used six distances (1–6) and distinguished between pairs that included zero and those that did not. In this study nonsymbolic zero was operationalized as an empty set (i.e., null numerosity). The results revealed that in the homogeneous condition, comparisons involving null numerosity (presented either as symbolic zero or an empty set) produced shorter RTs than pairs without zero, consistent with the end effect, and showed attenuated distance effects compared to other comparisons. In contrast, in the heterogeneous (mixed) block, comparisons between symbolic zero and dot arrays yielded a significant distance effect, whereas comparisons between an empty set and a digit did not. Accordingly, the authors concluded that pair format homogeneity is crucial. It allows the empty set to be perceived as a numerical entity. This ensures it is represented on the same scale (MNL) as non-zero numbers, much like symbolic zero.

Despite the importance of these findings, the studies by Lyons et al. (2012) and Zaks-Ohayon et al. (2022) have several limitations regarding the control of non-numerical magnitudes. In nonsymbolic tasks, physical properties such as total surface area, density, and visual features typically correlate with the number of items. When these non-numerical magnitudes are deliberately manipulated to be negatively correlated with the quantity—known as the incongruent condition—performance is typically slower and less accurate due to the conflict arising from the automatic processing of irrelevant visual features (e.g., Leibovich et al., 2017).

Lyons et al. (2012) specifically noted that such non-numerical visual properties may confound magnitude judgments, as groups containing larger or denser items are usually perceived as more numerous. These visual differences become particularly pronounced when null numerosity is represented by an empty set, which inherently lacks all continuous physical properties, creating an extreme perceptual contrast. When trials are designed to be incongruent, the task additionally engages cognitive inhibition. This inhibitory demand varies across formats: it is highest in pure nonsymbolic blocks due to the direct interference of continuous visual properties, and lowest in pure symbolic blocks where such cues are absent. These manipulations significantly affect response times (RTs), which is especially critical in the study of Lyons et al. (2012), where the confirmation of the primary hypothesis relied heavily on RT patterns.

Representing zero solely as an empty set reflects only one interpretation of zero, and this approach is problematic on two levels. First, on a perceptual level: RTs may be faster simply because it is visually easier to distinguish the presence of items from their complete absence. Second, on a conceptual level, zero is not always equivalent to an empty set. In real-world contexts, zero frequently denotes the absence of a particular element within a larger set rather than absolute nothingness. For instance, a refrigerator may be full but still contains zero milk cartons.

## The current study

The current study aims to investigate the cognitive representation of zero and its relationship with positive integers across different formats. The first research question investigates whether the processing of zero is format-dependent, extending the framework of Lyons et al.(2012), who demonstrated format-dependent processing for positive integers, to the domain of null numerosity. Specifically, we examine whether symbolic and nonsymbolic zeros are processed by the same underlying cognitive mechanism. The second research question explores whether zero behaves as a numerical point on the MNL or as a categorical boundary. To this end, we investigate how representational context and different numerical formats modulate the distance effect when zero is involved.

These questions enable us to determine whether zero is functionally integrated within the numerical magnitude system or processed as a conceptual outlier, and whether its cognitive status depends on representational format. In line with contemporary perspectives, we refer to the ‘mental number line’ as a useful descriptive framework for numerical organization rather than a literal spatial map.

**Fig. 1:**
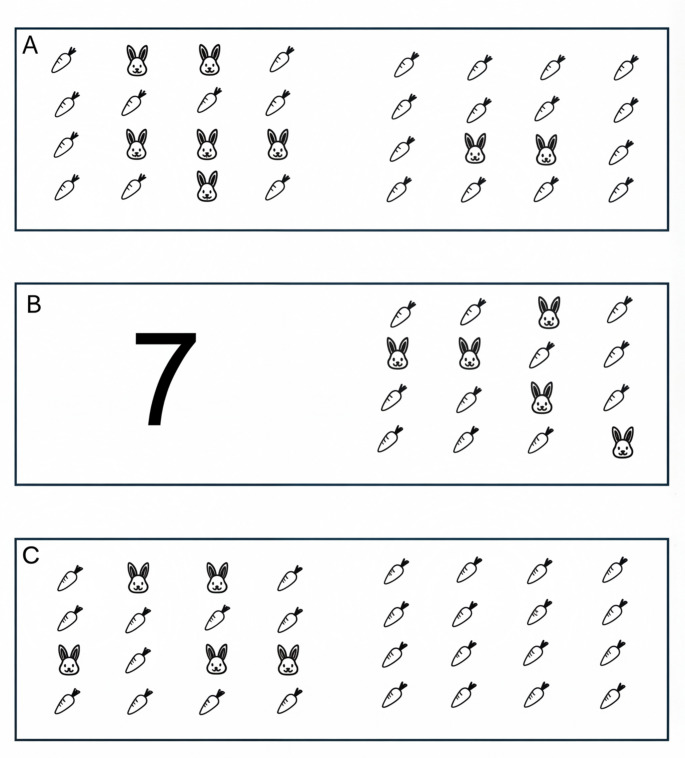
Stimuli. **A**. A homogeneous stimulus of two nonsymbolic quantities. **B**. A mixed stimulus of symbolic and nonsymbolic representations. **C**. A homogeneous stimulus of two nonsymbolic quantities, that includes nonsymbolic zero. The bunny is the task-relevant stimuli, and the carrot is the irrelevant stimuli

Our experimental design is inspired by Zaks-Ohayoun et al. (2022) (Exp. 1) while addressing some of its limitations. We controlled continuous magnitudes by placing our sets of objects on a 4 × 4 matrix containing both relevant and irrelevant items (Fig. 1). This achieved two goals. First, all the cells of the matrix were always full, either by the relevant or by the irrelevant items, creating only minimal differences in non-numerical magnitudes. Second, we expanded the use of zero from an empty set to a set empty of the task-relevant item, therefore providing a more ecological interpretation of nonsymbolic zero and reducing the physical difference from the empty set.

## Method

The task, the raw data and the analysis can be found in the following link: https://osf.io/gkpxa/overview?view_only=0366249443ba48d58fd24661e738042e.

### Participants

Using G*Power 3.1.9.2 (Kang, 2021), we calculated the sample size to be 50, for a three-way within-subjects ANOVA with an alpha of 0.05, power of 0.95, and an effect size of 0.15. Accordingly, the experiment included 50 neurotypical adults (31 females, 46 right-handed), aged 18–35 (Mean age = 28 years, SD = 5.3 years). All participants were recruited via social media. They provided digital consent. The study protocol was approved by the Faculty’s IRB. No participants were excluded.

### Stimuli

The stimuli were created using PowerPoint software and presented using OpenSesame software version 3.3.14 (Mathôt et al., 2012) on the participant’s private Windows computers. The symbolic stimuli (Arabic numerals) were presented as pairs of digits ranging from 0 to 9. The digits were presented in black Helvetica font with a height of 96 px inside a white rectangle (388 × 218 px). The nonsymbolic stimuli sets consisted of a white 4 × 4 matrix (388 × 218 px), with a transparent border between the cells. Each cell contained an item (Fig. 1). The items were either icons of bunnies (task-relevant) or carrots (task-irrelevant). Nonsymbolic zero was represented as a matrix containing only carrots instead of an empty set. From each quantity (other than zero) there were six different arrangements of the bunnies relative to the carrots.

### Experimental conditions

Our independent variables were distance (1–6), inclusion of zero (with zero, without zero) and pair format (symbolic, nonsymbolic, mixed). Accordingly, we had 18 different pairs without zero and six different pairs with zero, for a total of 24 pairs. To equal the number of pairs with and without zero, we tripled the number of times that we introduced the pairs with zero, bringing the number of pairs to 36. Next, we doubled the number of all the pairs to counterbalance the stimuli, yielding 36 × 2 = 72 pairs. Next, we tripled this number to create the different format conditions for each pair (pure symbolic, pure nonsymbolic, mixed), resulting in 72 × 3 = 216 pairs. Finally, we created two repetitions of each pair, yielding 216 × 2 = 432 trials.

We had three blocks with all the above conditions in random order, one block with 153 trials, one block with 140 trials, and one block with 139 trials. Due to a technical error, only 95% of the trials in each block were run. However, since the order of the trials and their selection was random, we still had all the conditions in every block.

**Fig. 2:**
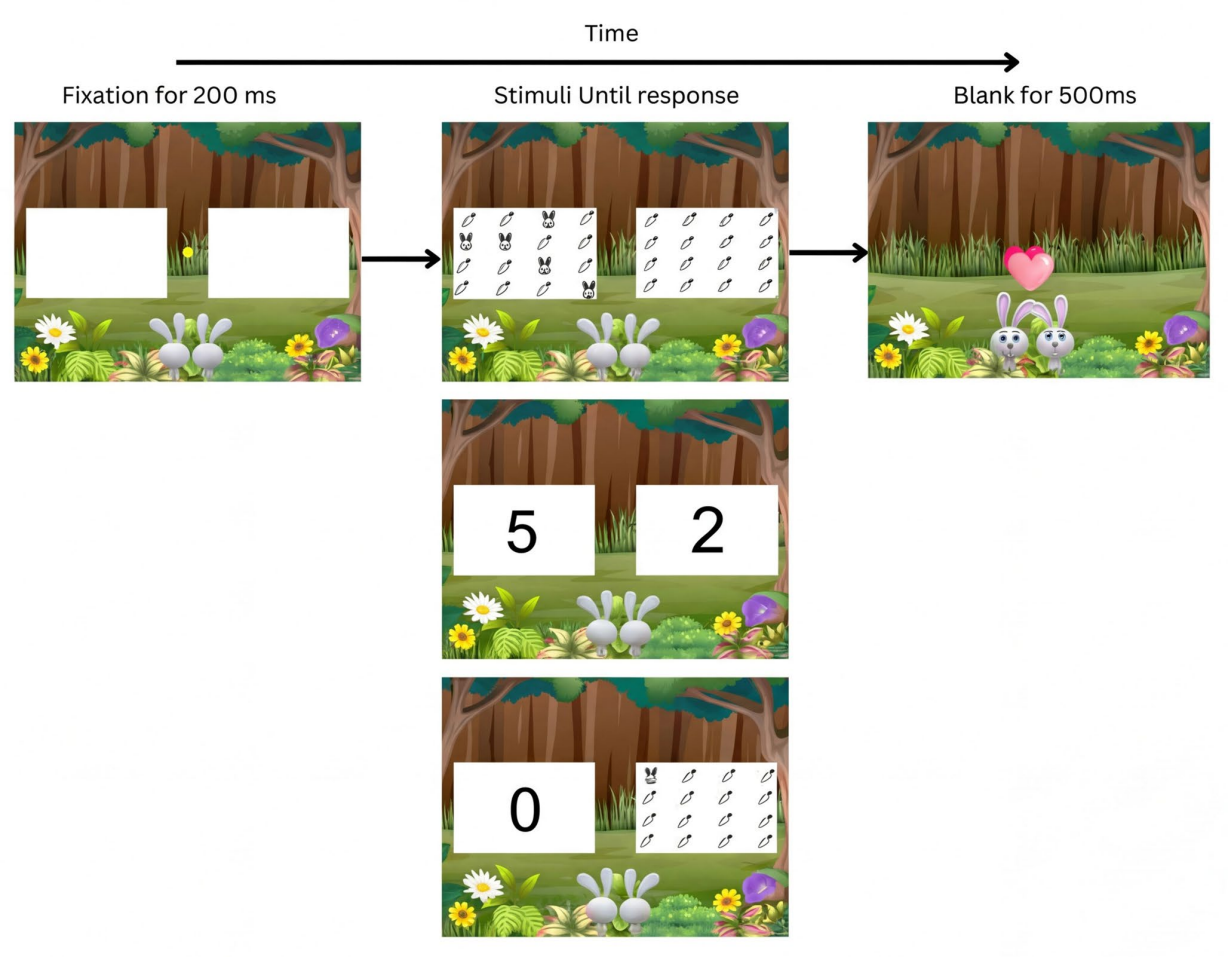
Experiment block procedure

### Procedure

The experiment was conducted via a Zoom meeting, with participants sharing their screens with the experimenter. Prior to the experiment, participants read and signed a consent form. Since this experiment was designed to be child friendly as well, we introduced the instructions as the following story: “Mommy and Daddy Bunnies lost their baby bunnies. Help them find them! On each screen, you’ll see bunnies on both sides, or a number that shows how many bunnies there are. Sometimes there will be a number on one side, and bunnies on the other. You need to choose which side has more bunnies, as quickly as you can without making mistakes. If there are more bunnies on the right side of the screen, press ‘J’. If there are more bunnies on the left side of the screen, press ‘F’.”

To ensure clear instructions and resolve any technical issues, a training session preceded the experimental blocks. Practice trials included feedback after each response. The practice ended when a participant achieved eight consecutive correct responses. The experimental blocks began next, consisting of three blocks with breaks between them.

The background for all the slides was a forest, to match the story in the instructions. In all the slides, except for the “long blank”, there were two white rectangles where the stimulus would appear. In each trial, a yellow fixation point appeared in the middle of the screen for 200 ms, followed by the target until response (“j” or “f”), and then a blank screen for 500 ms before a new trial began (Fig. 2).

## Results

Prior to analysis, outlier trials were removed using a two-stage process. First, trials with RTs below 200 ms or above 3000 ms were excluded. Second, for each participant and distance condition, trials exceeding 2.5 standard deviations (SD) from the individual mean were removed. This resulted in 2.4% of the correct trials being removed. Accuracy levels were high (Mean accuracy across trials = 98.1%, SD = 0.14). Therefore, we analyzed only RTs.

### RQ1: Format-dependent processing

To test for format-dependent processing, we performed within-factor two-way ANOVA with zero inclusion (yes/no) and pair format (symbolic, nonsymbolic, mixed) as the independent factors, and RT for correct responses as the dependent factor.

In this analysis, since the assumption of sphericity was violated for format type, we used Greenhouse-Geisser corrections where required. The analysis revealed a main effect for format type F (1.3, 96.9) = 264.1, *p* = 4.07 × 10^− 27^, $\:{\eta\:}_{p}^{2}=.84$. Further analysis shows that in general, RTs to symbolic pairs (M = 490ms, SD = 43ms) were faster than mixed pairs (M = 706ms, SD = 121ms; Cohen’s d = 2.44), that were faster than nonsymbolic pairs (M = 727ms, SD = 133ms; Cohen’s d = 0.55), all p_holm_ < 0.009.

In addition, there was also a main effect for zero inclusion F (1, 49) = 383, *p* = 8.29 × 10^− 25^, $\:{\eta\:}_{p}^{2}=.89$, suggesting that RTs to pairs with zero were generally faster than for pairs without zero.

**Fig. 3:**
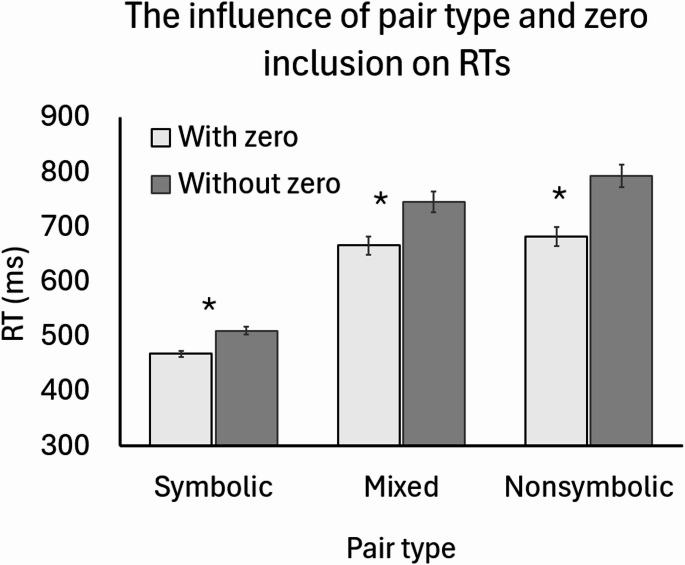
RT as a function of pair type and zero inclusion. The * indicates statistically significant differences

Importantly, there was also an interaction between format type and zero inclusion F (2, 98) = 57, *p* = 5.41 × 10^− 17^, $\:{\eta\:}_{p}^{2}=.54$. Post-hoc contrasts suggested that across all the different formats, the same pattern as in the main effects held. Namely, symbolic < mixed < nonsymbolic and without zero < zero. However, the differences between the symbolic pairs with and without zero are the smallest (d = 0.14), while in the nonsymbolic pairs this gap is the largest (d = 0.87) (all ps < 4.75 × 10^− 6^). The results are depicted in Fig. 3.

### RQ2: Mental number line integration

To fully address the integration of zero within the numerical continuum, we performed a three-way within-subjects ANOVA. This analysis subsumes the effects reported for RQ1 while allowing for a comprehensive examination of the interaction between format, zero inclusion, and numerical distance.

**Fig. 4:**
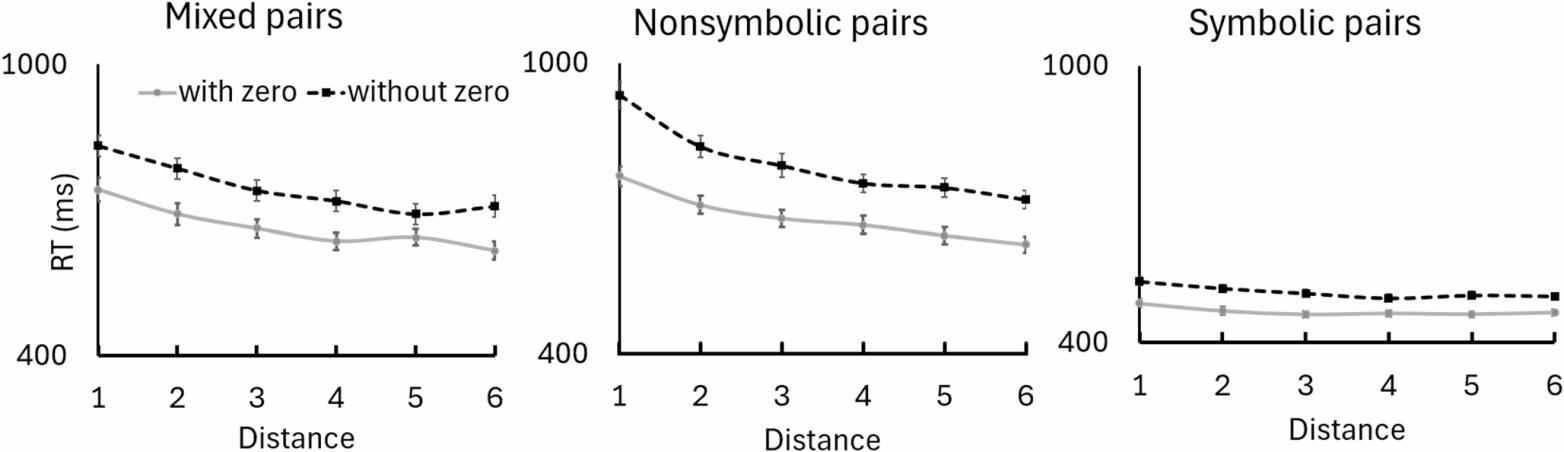
RT as a function of distance for different pair-types and zero inclusion. Based on raw RT data

Here, we focus on the main effect for distance F (3.1, 149.6) = 182.8, *p* = 3.11 × 10^− 17^, $\:{\eta\:}_{p}^{2}=.55$. All the interactions were also significant: between pair format and zero inclusion (as reported for RQ1), between pair format and distance F(6.98, 342.3) = 46.31, *p* = 2.93 × 10^− 6^, $\:{\eta\:}_{p}^{2}=.49$, between zero inclusion and distance F(3.68,180.2) = 8.9, *p* = 2.93 × 10^− 6^, $\:{\eta\:}_{p}^{2}=.15$, and between pair format, zero inclusion and distance F(7, 343.2) = 4.7, *p* = 4.7 × 10^− 5^, $\:{\eta\:}_{p}^{2}=.09$. Post-hoc comparisons confirmed that the distance effect was most robust in the nonsymbolic format without zero (e.g., distance 1 vs. 6, d = 1.54) compared to the symbolic format (d = 0.23, ns), and that zero inclusion provided a larger facilitation in the nonsymbolic (d = 0.87) than in the symbolic format (d = 0.14). RT as a function of pair format and zero inclusion is depicted in Fig. 4.

### Testing differences in the distance effect: Trend analysis and model comparison

Following the significant interaction effects identified in the ANOVA, we conducted additional analyses to characterize the functional relationship between distance and RT within each experimental condition. Specifically, two competing models were evaluated: a simple linear model and a power-function (nonlinear) model. The linear model assumes a direct linear relationship between distance and RT, whereas the power-function model allows for a more flexible, non-monotonic association, potentially capturing cognitive phenomena such as diminishing sensitivity or perceptual saturation at higher distances.

Given the limited number of observations per condition and substantial noise measurement, all parameter estimates were obtained using bootstrap resampling. Bootstrap resampling mitigates the effects of small sample sizes and violations of normality assumptions by generating empirical confidence intervals from the data (Efron & Tibshirani, 1998). For each participant and each condition (pair format and zero inclusion), a linear regression was fitted to RT as a function of distance. Separately, a log-log regression estimated the exponent *b* in the power-function RT = a x distance ^ b. Both sets of analyses were repeated across 1,000 bootstrap samples drawn with replacement from the pool of participants in each condition, yielding robust estimates of mean slope, exponent, and their respective 95% confidence intervals.

This analytical approach was chosen for two reasons. First, visual inspection of raw data suggested deviations from strict linearity, making it theoretically and empirically appropriate to compare both models. Second, the use of bootstrap methods and robust post-hoc tests ensured that findings were not unduly influenced by data sparsity or unequal variances, and that conclusions about model fit and group differences reflected genuine cognitive effects rather than analytical artifacts. The MATLAB code and the raw data can be found in the data repository (see link at the beginning of the method section).

To formally test the nature of the distance effect, one-sample t-tests were conducted on the bootstrapped exponents (b) to determine if they significantly differed from 1 (the value expected under a strictly linear relationship on the original scale). Further, a one-way repeated-measures ANOVA was performed across all six conditions to evaluate whether exponent values differed between conditions. Where the ANOVA was significant, pairwise post-hoc comparisons were conducted with Tukey correction to control family-wise error rates, thus reducing the risk of type I error due to multiple comparisons.

The trend analysis revealed that, across all experimental conditions, the linear slope relating RT to distance was negative, indicating that RT consistently decreased as numerical distance increased. For all six conditions, bootstrap-derived 95% confidence intervals for the linear slope excluded zero, demonstrating robust and significant distance effects in each condition (Table 1; Fig. 4).

**Table 1:** Summary of linear and power-law fits for each condition: Means and bootstrap 95% confidence intervals for the linear RT-distance slopes and power exponents

Condition	Mean Linear Slope	95% CI Slope	Mean Power Exponent (b)	95% CI (b)	t (b vs. 1)	*p* (b vs. 1)	*R*^2^ (power)	*R*^2^ (linear)
Mixed With Zero	−22.67	[−25.45, −19.97]	−0.1	[−0.11, −0.09]	−195.27	< 1e-70	0.98	0.88
Mixed Without Zero	−26.43	[−30.88, −21.60]	−0.1	[−0.12, −0.08]	−137.07	< 1e-64	0.96	0.86
Nonsymbolic With Zero	−25.97	[−28.72, −23.35]	−0.11	[−0.12, −0.10]	−202.96	< 1e-73	0.99	0.93
Nonsymbolic Without Zero	−39.25	[−44.59, −34.08]	−0.14	[−0.16, −0.12]	−133.43	< 1e-64	1.00	0.87
Symbolic With Zero	−3.5	[−5.62, −1.39]	−0.02	[−0.04, −0.01]	−170.24	< 1e-69	0.72	0.48
Symbolic Without Zero	−6.14	[−7.94, −4.45]	−0.04	[−0.05, −0.03]	−211.92	< 1e-74	0.93	0.73

Comparison of model fits indicated that a power function provided a better account of the data than a strictly linear trend. As shown in Table 1, the power function consistently yielded higher R^2^ values compared to the linear model.

Crucially, the improvement in fit was most pronounced in the symbolic condition with zero, where R^2^ increased from.48 (linear) to.72 (power), representing a substantial 50% increase in explained variance. In contrast, improvements in the nonsymbolic conditions were more modest (e.g., from 0.93 to 0.99 for nonsymbolic with zero), suggesting that the power function captures a specific non-linear psychophysical behavior rather than merely benefiting from additional degrees of freedom.

The exponent parameter b from the power model was negative and close to zero for all conditions, and in all cases, differing significantly from 1 (all p_Tukey_ <10^− 64^), indicating strong non-linearity. This non-linear pattern was primarily driven by a pronounced drop in RT from distance 1 to 2, with more gradual decreases at higher distances (Fig. 4).

Direct comparisons of the exponents across conditions using ANOVA and post-hoc tests revealed that the exponent did not significantly differ between Mixed pairs with and without zero (mean diff = −0.025, 95% CI [0.0015, 0.028], p_Tukey_ = 1.00), nor between Symbolic pairs (mean diff = −0.015, 95% CI [0.012, 0.038], p_Tukey_ = 0.81). However, within the Nonsymbolic condition, the exponent was significantly more negative for pairs without zero compared to those with zero (mean diff = 0.006, 95% CI [0.0325, 0.059], p_Tukey_ = 0.006), indicating a slightly but reliably steeper non-linearity when zero was not included. Namely, the end effect, a reduction in the distance effect, was evident only for the nonsymbolic pairs.

### Analysis of endpoint facilitation

To further investigate the unique role of zero and one as numerical endpoints, we examined the distance effect for each endpoint condition (0, 1, or none) across formats. Individual slopes were calculated using bootstrap analysis (5,000 iterations) to determine the stability of the distance effect when anchored by these specific values.

**Fig. 5:**
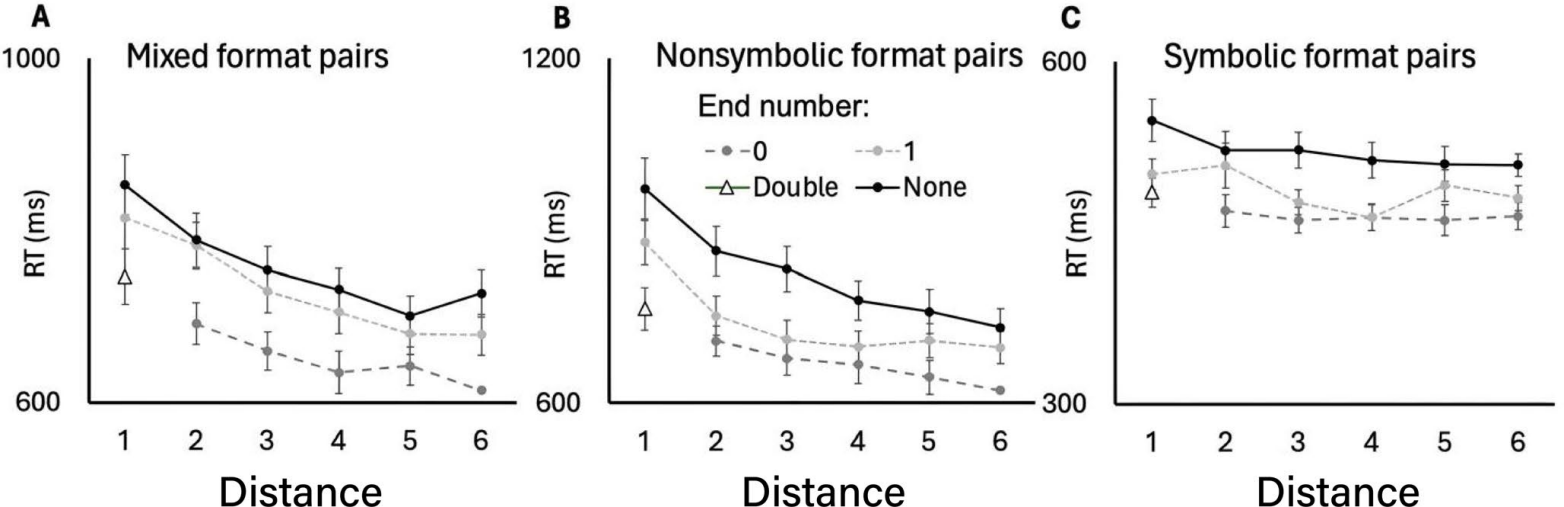
RT as a function of distance for different pair-types and end-types. Based on raw RT data

A summary of these comparisons revealed that the presence of an endpoint significantly facilitated processing speed, particularly when zero was involved (see Table 2; Fig. 5). Bootstrap analysis revealed shallower distance-effect slopes for zero-anchored pairs in these nonsymbolic and mixed formats, indicating a unique categorical facilitation (Fig. 5). Crucially, the [0, 1] pair emerged as the fastest condition for these formats, representing a “disproportionate jump” that stands in contrast to the continuous trends observed in previous studies (Fig. 5b).

In the Symbolic format, however, this distinction between zero and one endpoints was not statistically significant, suggesting a more integrated processing of numerical magnitudes in this notation. Detailed statistical results for all bootstrap comparisons and format interactions are summarized in Table 2.

**Table 2:** Significance tests for distance effect in different conditions

Pair format	End Number	mean slope	CI lower	CI upper	*P* value
Symbolic	0	−0.7	−2.9	1.5	0.5516
	None	−3.4	−6.5	−0.6	0.0172
	1	−4.5	−8.5	−0.6	0.0212
	0	−17.2	−21.2	−13.4	0.000001
Mixed	None	−19.0	−24.6	−13.6	0.000001
	1	−23.5	−29.4	−17.8	0.000001
	0	−20.1	−23.0	−17.4	0.000001
Nonsymbolic	None	−33.2	−38.6	−27.6	0.000001
	1	−14.1	−21.6	−7.2	0.0004

## Discussion

The present findings shed new light on how the representation of zero interacts with fundamental magnitude comparison processes. Across symbolic, mixed, and nonsymbolic formats, the numerical distance effect showed a distinctly nonlinear profile, characterized by a steep decline in RTs from small to medium distances followed by a gradual plateau. However, this pattern varied systematically across formats. In the **symbolic format**, the distance effect was reliable only when endpoint pairs (0 or 1) were included and disappeared when these boundary values were removed, suggesting that symbolic comparisons may depend more strongly on the presence of salient anchors. In the mixed format, distance sensitivity remained relatively stable regardless of whether endpoint pairs were included, indicating consistent performance across notations. In the **nonsymbolic format**, the distance effect was generally weaker for endpoint pairs than for mid-range pairs, yet similar for 0 and 1 as lower anchors. Finally, for the double-endpoint pair (0–1), responses were faster than for equidistant pairs with a single or no endpoint in the mixed and nonsymbolic formats, but not in the symbolic one. Collectively, these results demonstrate that both the magnitude and the shape of the distance effect vary across representational formats and depend on whether zero serves as a categorical boundary within the comparison set.

In what follows, we discuss the implications of these findings in three complementary parts: first, how the distance pattern changes when zero enters the system; second, how the methodological design constrains theoretical interpretation; and finally, how these aspects converge toward a unified account of zero representation.

### The distance effect and the semantic boundary of zero

In the symbolic condition, RTs followed a non-linear distance pattern with pronounced facilitation for pairs involving zero. Once endpoint values were removed, the distance effect vanished entirely. Whereas prior studies reported a robust distance effect for comparisons involving 0 (e.g., Zaks-Ohayon et al., 2022), in our data the effect appeared only when endpoint pairs (0 or 1) were included and disappeared when these pairs were excluded.

Interestingly, our results in the symbolic format contrast with those of Varma and Schwartz (2011). While they found that including zero in a range of negative and positive numbers eliminated the distance effect for zero-positive pairs, our data showed that in a single-digit positive range, the presence of zero (or one) was the primary driver of the distance effect. This discrepancy suggests that the cognitive status of zero is highly context dependent. In our study’s context, where zero defines the absolute lower boundary of the set, it functions as a salient anchor that sharpens semantic contrasts. Conversely, in a broader context involving negative numbers, zero may lose this unique anchoring function. This pattern indicates that, in the symbolic domain, for positive numbers, boundary values drive rather than diminish sensitivity to numerical distance—a result not previously documented and suggestive of a distinct underlying mechanism.

Classic findings support the idea that zero engages representational processes that differ from those used for positive integers. In a large-scale number naming study, Brysbaert (1995) showed that RTs for numbers 1–99 were well explained by a logarithmic–frequency function combined with phonological factors, yet zero systematically deviated from this pattern, yielding slower responses than expected. This deviation does not imply that zero lies outside the magnitude system; rather, it suggests that zero activates semantic processes not reducible to graded quantity, consistent with its role as the conceptual marker of absence.

This interpretation converges with symbolic-comparison findings showing that distance effects do not always reflect graded quantity differences (Krajcsi & Kojouharova, 2017). In their artificial-notation experiment, participants learned symbols for values 1–3 and 7–9 while the middle range was omitted. After confirming full mastery of the notation, the authors found that comparisons crossing the gap (e.g., 3 vs. 7) behaved as if the distance were a single unit rather than four. They concluded that symbolic distance effects reflect learned “small–large” associations rather than true numerical spacing—a view formalized in the Discrete Semantic System (DSS) model, which characterizes symbolic numbers as nodes in a conceptual network linked by semantic relations rather than by metric distances. This suggests that symbolic zero functions as a distinct category rather than a simple point on a linear continuum.

Our statistical findings reinforce this distinction between linear metric spacing and non-linear anchoring. Specifically, the substantially higher R^2^ values for the power function, most notably in the symbolic condition with zero, where explained variance increased by 50% indicate that zero-related comparisons follow specific non-linear psychophysical patterns. These statistical results suggest that while zero serves as a semantic anchor, its integration into the magnitude system reflects a complex, non-monotonic association rather than a simple linear progression.

The distinct facilitation observed for zero-anchored pairs also allows us to differentiate our findings from a simple ‘size effect’, the classic phenomenon where for the same distance, comparing smaller numbers is faster than comparing larger ones. In Parkman’s (1971) linear counting model, RTs are primarily a function of the smallest digit in a pair. However, our results diverge from this model in two critical ways. First, while Parkman noted that zero actually increased difficulty and RTs in his task, our participants showed significant facilitation for pairs containing zero. Second, a continuous size effect cannot account for the ‘disproportionate jump’ in performance observed for the [0, 1] pair in mixed and nonsymbolic formats, which was significantly faster than the linear trend established by pairs with distance one like [1, 2] or [2, 3] would predict. In addition, for the symbolic format, where the size effect would predict faster RTs for [0, 1], we found no significant RT difference between pairs with distance one and the pair [0, 1]. This supports our proposal that zero triggers a categorical boundary process rather than a standard analog magnitude comparison. In the symbolic domain, this process is driven by specific semantic markers rather than metric quantities.

The same logic accounts for our symbolic pattern. If symbolic distance effects arise from contrasts in learned “small–large” associations, then endpoint digits such as 0 and 1 carry disproportionately strong “smallest” tags that sharpen these contrasts. Consequently, a distance effect emerges only when these endpoints are present, whereas pairs drawn from the interior of the range (2–9) lack stable semantic spacing and therefore do not yield a graded distance pattern.

Within this discrete–semantic framework, zero naturally occupies a unique position because it represents the absence of physical stimulation, in contrast to positive integers which denote the presence of quantity. This fundamental distinction marks the transition from ‘none’ to ‘some’ as a categorical shift rather than a mere metric difference. This perspective also explains why DSS principles applied selectively to our symbolic condition, whereas the nonsymbolic and mixed formats continued to produce regular distance effects, reflecting a greater reliance on analog quantity representations.

Consistent with this interpretation, Pinhas and Tzelgov (2012) demonstrated that zero functions as an end-anchor on the MNL, suggesting that “0, or 1 in the absence of 0, is perceived as the smallest entity” (p. 1187). They concluded that “while ‘1’ acts as an inherent anchor, ‘0’ is a culturally acquired symbolic boundary marking the transition from none to some” (see also Nieder, 2016).

Together, these results highlight that symbolic numerical processing is guided by semantic organization rather than by analog magnitude alone. By redefining the roles of 0 and 1 as semantic anchors, we demonstrate that zero serves as a meaningful structural boundary that dictates the relational organization of the symbolic system. The conclusion regarding zero aligns with recent evidence suggesting that such magnitude-based structural anchors are a fundamental feature of semantic organization even beyond the numerical domain. In a recent study by Varma et al. (2024) adult participants judged which of two words had “more” or “less” of a property. Comparisons of words straddling a boundary (e.g., *cold* vs. *warm* on a temperature scale) were faster than comparisons of words at the same levels on scales without a boundary (e.g., *some* vs. *most* on a Quantity scale).

### The methodological design as theoretical evidence

Our methodological choices were made not only to ensure experimental rigor but also to inform the theoretical interpretation of the results. The design was constructed to reduce perceptual confounds and strategic variability, allowing us to isolate the cognitive and semantic sources of the observed effects. Three aspects of the paradigm are particularly important in this regard.

First, we aimed to minimize differences in continuous visual magnitudes that typically correlate with numerosity, such as total surface area, density, and convex hull. To do so, we constructed all stimuli as fully occupied matrices, so that both the relevant and irrelevant elements were evenly distributed across space. This approach ensured that potential differences in low-level visual properties were minimal and unlikely to reach perceptual salience. Importantly, unlike studies that attempt to “neutralize” continuous magnitudes by presenting an equal number of congruent and incongruent trials, an approach that does not truly eliminate perceptual influences (for a review see Leibovich et al., 2017), our design reduced these differences at their source by avoiding any systematic correlation between numerosity and visual cues. Therefore, we can infer that the observed distance patterns rely less on low-level perceptual features and more on cognitive magnitude processing.

Second, we represented zero using a set with zero relevant stimuli. This choice was crucial because it separated the semantic meaning of “zero” from the mere absence of stimulation. Previous work has shown that perceiving an empty set as a numerical entity depends heavily on task context and format homogeneity. For example, Merritt et al. (2009) demonstrated that rhesus monkeys treated an empty set as a numerical value only when it was presented as the smallest quantity within a structured numerical range. Under these conditions, their choices showed a reliable distance effect, indicating genuine magnitude-based comparison between zero and non-zero quantities. However, when the empty set appeared without such numerical context, performance became inconsistent and no longer resembled numerical processing. Merritt and Brannon (2013) reported a similar pattern in preschool children. At the neural level, Okuyama et al. (2015) and Ramirez-Cardenas et al. (2016) identified parietal neurons selectively tuned to “zero” versus “non-zero” numerosities, indicating that null numerosity is encoded as a distinct category rather than simple perceptual absence. Finally, Zaks-Ohayon et al. (2022) demonstrated that an empty set is interpreted as “zero” only under homogeneous conditions, not when formats are mixed.

Building on this evidence, we extended the logic of the ‘empty set’ by introducing visually present but task-irrelevant elements across all stimuli. By holding attention, visual context, and perceptual load constant, we ensured that ‘zero’ was interpreted as a numerical boundary within a meaningful scene, rather than as the mere absence of a scene altogether. This design choice was pivotal: it shifted the task from a perceptual detection of ‘nothing’ to a conceptual, category-based processing of ‘zero’. Consequently, our findings provide a more direct assessment of zero’s semantic role, demonstrating that its function as a structural boundary is an inherent property of the numerical system rather than a byproduct of perceptual salience or visual contrast.

Third, we presented mixed, random-order trials requiring participants to maintain consistent cognitive control across conditions, and dealing with two types of stimuli: relevant and irrelevant. Importantly, our mixed condition produced the opposite pattern to that reported by Lyons et al.(2012): mixed pairs were not slower than homogeneous symbolic or nonsymbolic pairs. This finding suggests that under the current conditions of sustained cognitive control and a shared visual context, symbolic and nonsymbolic information may be processed within a unified relational system, rather than through separate representational codes. However, because RTs can also reflect control demands, attentional shifts, or strategy selection, caution is warranted when drawing broad theoretical conclusions about shared representations based on response latencies alone.

### Integration: Toward a unified account of zero representation

Taken together, the present findings suggest that zero does not merely extend the numerical continuum but transforms its structure. Rather than functioning as another point on a mental scale, zero appears to redefine the system’s boundaries. It marks a qualitative shift—from absence to quantity, from “nothing” to “something”—and thus creates the conceptual ground on which numerical meaning itself can unfold.

This pattern was confined to the symbolic format, where zero acted as a semantic boundary that organizes the structure of the numerical system. In contrast, nonsymbolic quantities retained graded relations even when contextual anchors were absent, suggesting that only symbolic representation relies on categorical rather than metric coding. This interpretation aligns with Krajcsi and Kojouharova’s (2017) proposal that the symbolic distance effect reflects associative and relational processing within a discrete semantic system, rather than continuous magnitude overlap—a mechanism that need not apply to nonsymbolic numerosity. Extending this idea, the present results imply that the symbolic system encodes not just “how much,” but where the concept of number begins.

The behavior of the [0, 1] pair further supports this format-dependent role. In nonsymbolic and mixed formats, this pair elicited significantly faster responses, reflecting a salient perceptual shift from absence to presence. Conversely, in the symbolic format, the [0, 1] pair did not show such unique facilitation relative to other pairs involving zero. This reinforces the view that in the symbolic system, zero functions primarily as a structural anchor that defines the scale, rather than a perceptual category that triggers specific facilitation.

Conceptually, these findings invite a reframing of the MNL itself. Rather than a literal spatial continuum, the MNL may be better understood as a dynamic semantic map whose structure depends on context, format, and task demands. Within this map, zero defines the lower categorical limit, the boundary that separates the realm of quantities from the conceptual notion of nothingness. This interpretation resonates with the Discrete Semantic System framework (Krajcsi & Kojouharova, 2017) and with current relational and semantic models of number representation, which view numerical symbols not as spatial coordinates but as flexible constructs shaped by meaning and use.

### Limitations

Two methodological limitations should be acknowledged. First, when estimating the distance effect at the individual level, the data were not sufficiently stable across participants to allow for reliable parametric fitting. To address this, we employed a bootstrap-based approach, which provides a robust estimation method and is well anchored in prior work using similar variability-sensitive measures (see Method). Second, the analysis focusing on the [0, 1] pair was conducted post hoc and included a relatively small number of data points. Although the observed pattern was theoretically consistent and statistically reliable, it should be interpreted cautiously until replicated in a study specifically designed to examine this contrast.

## General summary

Taken together, the present findings indicate that zero holds a distinct yet systematically integrated place within the numerical system. Across symbolic and nonsymbolic formats, its processing revealed both categorical and magnitude-based components, marking zero as the boundary between “none” and “some” rather than an element outside the continuum. By demonstrating this pattern within a unified paradigm, the study provides the first direct behavioral evidence that zero is cognitively represented as part of the magnitude system while preserving its unique semantic status—offering a refined view of how numerical meaning extends to the very notion of nothingness.

## Data Availability

All data supporting the findings of this study are available in the following OSF link: https://osf.io/gkpxa/overview?view_only=0366249443ba48d58fd24661e738042e.‎.
